# Change in the use of diagnostic tests in the management of lower respiratory tract infections: a register-based study in primary care

**DOI:** 10.3399/bjgpopen20X101015

**Published:** 2020-03-04

**Authors:** Anna B Moberg, Olof Cronberg, Magnus Falk, Katarina Hedin

**Affiliations:** 1 GP, Kärna Primary Healthcare Center and Department of Health Medicine and Caring Sciences, Linköping University, Linköping, Sweden; 2 Doctoral Student, Department of Health Medicine and Caring Sciences, Linköping University, Linköping, Sweden; 3 GP, Växjöhälsan Primary Healthcare Center and Department of Research and Development, Region Kronoberg, Växjö, Sweden; 4 Doctoral Student, Department of Clinical Sciences in Malmö, Family Medicine, Lund University, Malmö, Sweden; 5 Associate Professor, Department of Health Medicine and Caring Sciences, Linköping University, Linköping, Sweden; 6 Associate Professor, Department of Clinical Sciences Malmö, Family Medicine, Lund University, Malmö, Sweden; 7 GP, Futurum, Region Jönköping County, and Department of Health Medicine and Caring Sciences, Linköping University, Linköping, Sweden

**Keywords:** community-acquired pneumonia, primary care, management, C-reactive protein, chest X-ray, antibiotics, anti-bacterial agents

## Abstract

**Background:**

Differentiating between pneumonia and acute bronchitis is often difficult in primary care. There is no consensus regarding clinical decision rules for pneumonia, and guidelines differ between countries. Use of diagnostic tests and change of management over time is not known.

**Aim:**

To calculate the proportion of diagnostic tests in the management of lower respiratory tract infections (LRTIs) in a low antibiotic prescribing country, and to evaluate if the use and prescription pattern has changed over time.

**Design & setting:**

A register-based study on data from electronic health records from January 2006 to December 2014 in the Kronoberg county of south east Sweden.

**Method:**

Data regarding use of C-reactive protein (CRP), chest x-rays (CXRs), microbiological tests, and antibiotic prescriptions were assessed for patients aged 18–79 years, with the diagnosis pneumonia, acute bronchitis, or cough.

**Results:**

A total of 54 229 sickness episodes were analysed. Use of CRP increased during the study period from 61.3% to 77.5% for patients with pneumonia (*P*<0.001), and from 53.4% to 65.7% for patients with acute bronchitis (*P*<0.001). Use of CXR increased for patients with acute bronchitis from 3.1% to 5.1% (*P*<0.001). Use of microbiological tests increased for patients with pneumonia, from 1.8% to 5.1% (*P*<0.001). The antibiotic prescription rate decreased from 18.6 to 8.2 per 1000 inhabitants per year for patients with acute bronchitis, but did not change for patients with pneumonia.

**Conclusion:**

Use of CRP and microbiological tests in the diagnostics of LRTIs increased despite the fact that the incidence of pneumonia and acute bronchitis was stable.

## How this fits in

There are no consistent clinical decision rules for pneumonia, and guidelines regarding assessment differ between countries. Use of CRP and microbiological tests appears to be increasing in Sweden, a country with a low antibiotic prescription rate. During the same period there has been a significant reduction in antibiotics prescribed for acute bronchitis, indicating improved adherence to treatment recommendations. This emphasises the use of diagnostic testing as a piece of the puzzle in the management of lower respiratory tract infections (LRTIs).

## Introduction

Diagnosis of pneumonia is a challenge for primary care physicians since there are no sharply defined clinical criteria for the diagnosis. Several efforts have been made to identify a decision rule, but results vary.^1–5^ Guidelines and clinical decision rules on how to assess pneumonia in primary care differ between countries. Despite moderate sensitivity and specificity, CXR is the gold standard for the diagnosis of pneumonia.^1–6^ Some guidelines recommend CXR in the initial judgment, and others recommend CRP as a complement to clinical examination.^7–9^

Swedish criteria for possible pneumonia^6^ are: generally ill patient with tachypnoea (>20/min), tachycardia (>120/min), and symptoms such as fever, cough, newly expressed fatigue, and lateralised breath pain. Common findings are focally depressed or altered breathing sounds (crackles or wheezes), or dullness to percussion. CXR is not recommended in the initial judgment, nor is CRP testing. CRP can be considered when clinical diagnosis of LRTIs is unclear. Culture with resistance determination from sputum or nasopharyngeal swabs can be valuable when pneumonia is presumed, especially if the patient has been in an area with a high prevalence of bacterial resistance to antibiotics.^6^

A European study by van Vugt *et al* found that CRP >30 mg/L in conjunction with clinical examination refined the diagnostic information.^10^ Previous studies have shown that CRP is widely used in this manner in Scandinavia, but not to the same extent in other countries.^11,12^ Compared to most other countries, Sweden, the Netherlands, and a few other nations, have a low antibiotic prescription rate and low prevalence of antibiotic resistance in *Streptococcus pneumonia*e*,* the most common cause of pneumonia.^13,14^ The drug of choice to treat pneumonia in Sweden is phenoxymethylpenicillin, followed by doxycycline.^6^ To the authors’ knowledge, if or how the management of LRTIs has changed over time has not been explored in a low prescribing country.

The aim of the present study was to calculate the proportion of CRP, CXR, and microbiological tests used in the management of LRTIs in Swedish primary care, and to evaluate if the use had changed over a period of 9 years. The secondary aim was to investigate whether the extent and pattern of antibiotic prescriptions for LRTIs had changed over the same period.

## Method

### Design

This is a descriptive register-based study on data from electronic health records (EHRs) between January 2006 and December 2014. Data from the EHRs are routinely transmitted to a database separate from the records. All data were extracted on one occasion. Data contained information on patients diagnosed with acute bronchitis, pneumonia, or cough, who consulted primary care in the Kronoberg county of Sweden between January 2006 and December 2014. Information on radiology and microbiological tests was not available in the EHRs before 2008.

### Study population

**Figure 1.**
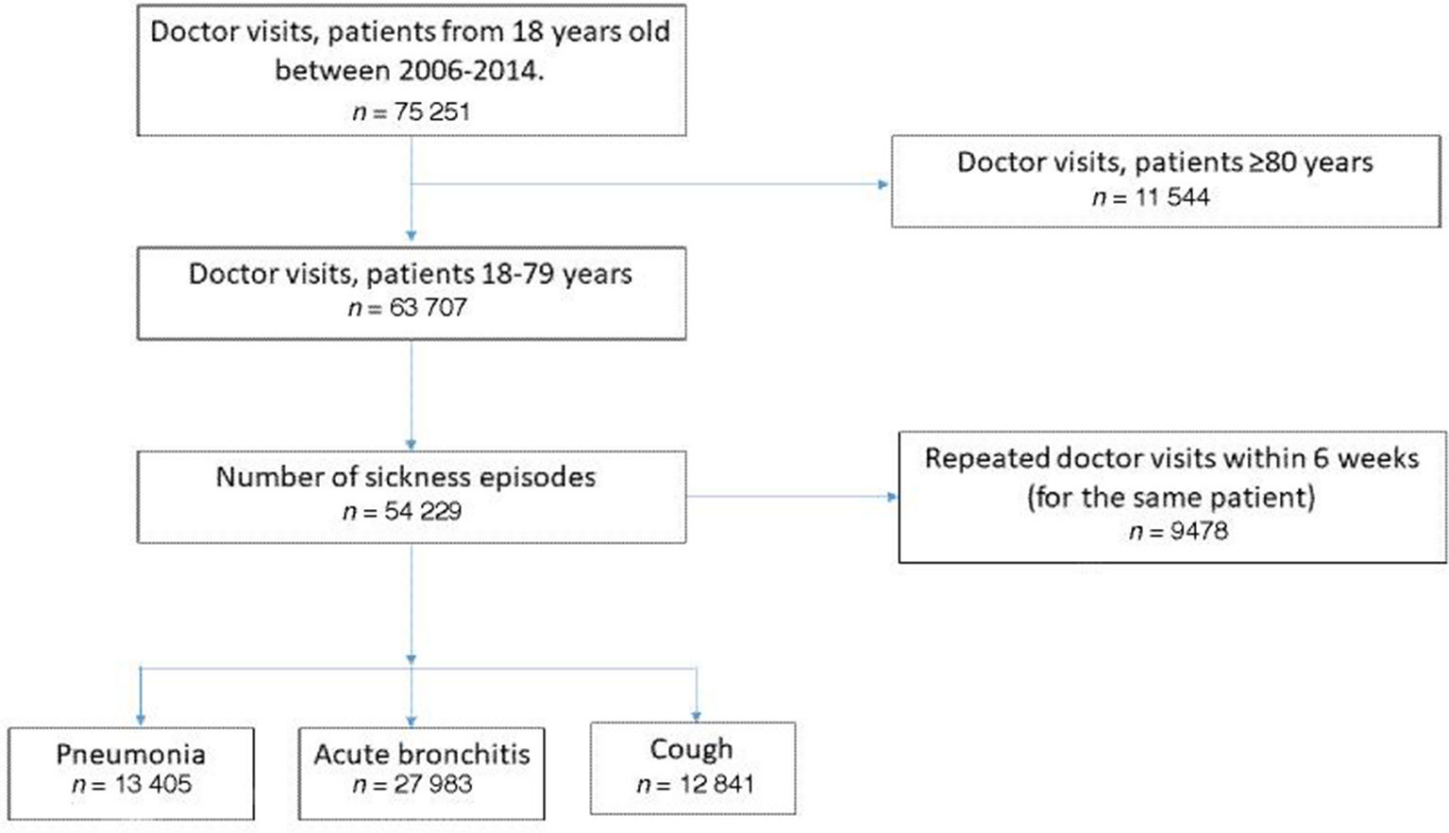
Flowchart of the inclusion and exclusion process

All primary health care centres (PHCCs) in Kronoberg participated. In total, 33 PHCCs and three out-of-hours offices were included in the study and provided data through the register. Adult patients with an LRTI — pneumonia (International Classification of Diseases and Related Health Problems version 10 [ICD-10] identifier: J18.-P); acute bronchitis (ICD-10 identifier: J22.-P); and the symptom diagnosis ‘cough’ (ICD-10 identifier: R05.-) — were eligible for analyses. Only data from consultations for patients aged 18–79 years were included. Due to some older patients receiving dose-dispensed medications administered through a computer system without connection to the EHR, patients aged >79 years were excluded for further analyses. Contacts occurring within 6 weeks for the same patient and diagnosis were considered to be one contact (Figure 1).

### Study variables

For each consultation the patient register provided information on sex, age at the consultation, PHCC, date, diagnoses, executed CRP tests, microbiological tests, radiography, and results of the tests performed. Information on any antibiotic prescription was also included. The Swedish primary health care version of the ICD-10 was used to identify the diagnoses.

### Data analyses

Diagnoses were ranked so that the diagnosis most likely to result in an antibiotic prescription received the highest rank (pneumonia), followed by acute bronchitis and cough. Thus, if patients were diagnosed with both cough and pneumonia or acute bronchitis, cough was removed since it was considered less serious. As different devices for CRP were used at different PHCCs, the CRP values were adjusted to 8–160 mg/L (values <8 mg/L were set to 8 mg/L and values >160 mg/L were set to 160 mg/L). Proportions and medians were calculated for descriptive data. A binary logistic regression model was used to analyse any significant change over time, using the first year as reference. When adjusting for confounders, a multiple logistic regression model was used. Pearson’s χ² test was used when analysing any difference in proportions. Mann-Whitney U test was used to identify any differences in skewed data. All statistical analyses were performed using IBM SPSS Statistics (version 23). *P* values <0.05 were considered significant.

## Results

In total there were 75 251 visits. The number of visits excluded is presented in Figure 1. After excluding patients aged ≥80 years and revisits within 6 weeks, 54 229 sickness episodes remained eligible for analyses. Among these, the median age was 55 years and 57.7% were female. Other characteristics and use of diagnostic tests are presented in Table 1.

**Table 1.** Characteristics of patients aged 18–79 years with lower respiratory tract infections in primary care, distribution of diagnostic tests performed, and proportion of patients prescribed antibiotics for each diagnosis

	**Total** **(*n* = 54 229**),*n* (%)	**Pneumonia** **(*n* = 13 405**),*n* (%)	**Acute** **bronchitis** **(*n* = 27 983**),*n* (%)	**Cough** **(*n* = 12 841**),*n* (%)
Median age, years	55	56	54	54
Median CRP value, g/L	14	62	11	8
Female	31 268 (57.7)	7066 (52.7)	16 890 (60.4)	7312 (56.9)
**Tests performed**				
CRP	33 254 (61.3)	9566 (71.4)	17 315 (61.9)	6373 (49.6)
CXR	4237 (7.8)	1657 (12.4)	1047 (3.7)	1533 (11.9)
Microbiology	1854 (3.4)	535 (4.0)	703 (2.5)	616 (4.8)
**Antibiotic prescription**	28 833 (53.2)	11 298 (84.3)	16 009 (57.2)	1526 (11.9)
Phenoxymethylpenicillin	8128 (15.0)	4577 (34.1)	3173 (11.3)	378 (2.9)
Doxycycline	15 954 (29.4)	4909 (36.6)	10 200 (36.5)	845 (6.6)
Amoxicillin	2469 (4.6)	832 (6.2)	1553 (5.5)	84 (0.7)
Erythromycin	1084 (2.0)	529 (3.9)	490 (1.8)	65 (0.5)
Cefadroxil	291 (0.5)	132 (1.0)	128 (0.5)	31 (0.2)
Others	906 (1.7)	318 (2.4)	465 (1.7)	123 (1.0)

CRP = C-reactive protein. CXR = chest x-ray.

**Figure 2.**
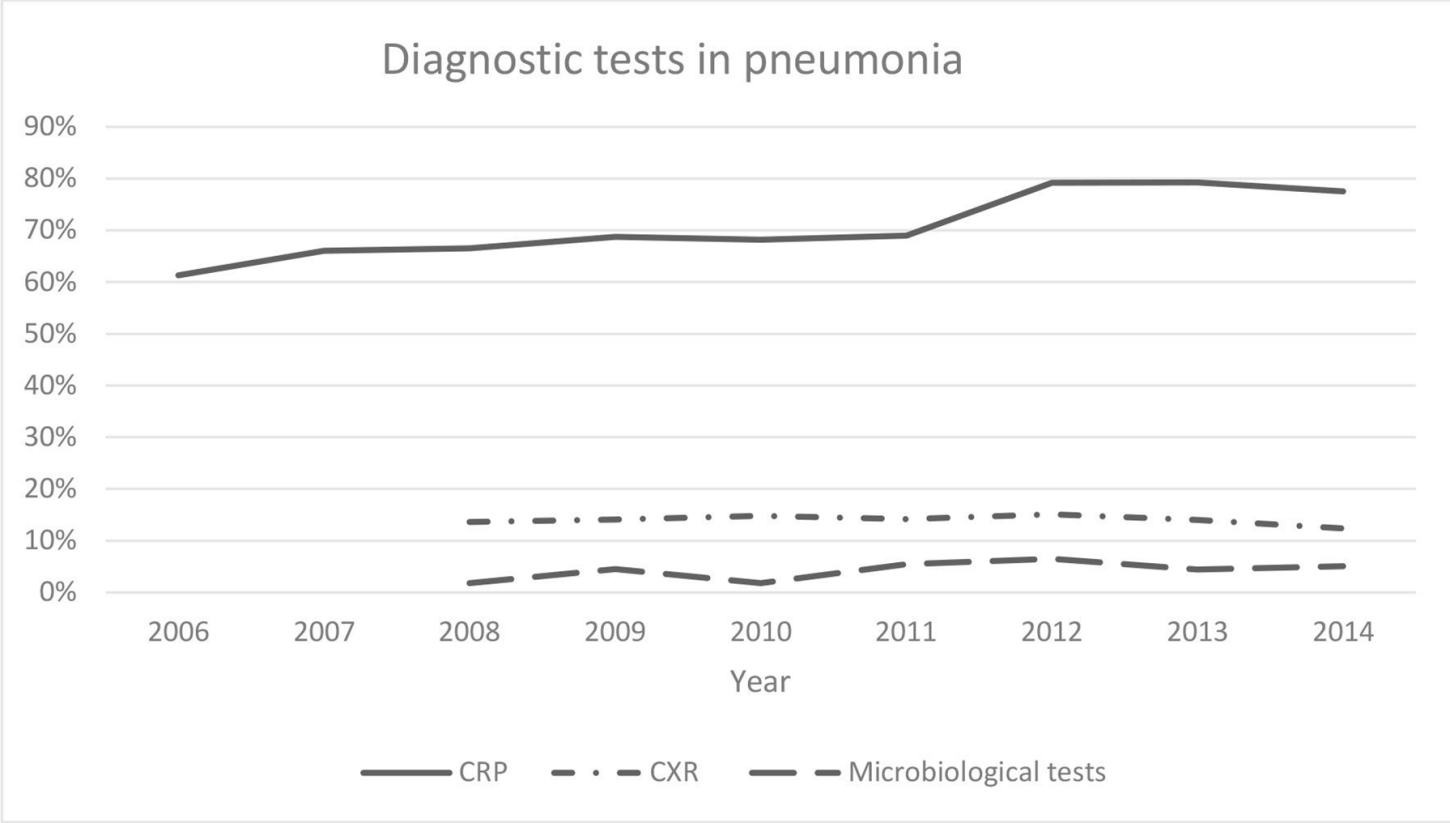
Proportion of patients aged 18–79 years with pneumonia in primary care, where C-reactive protein (CRP), chest x-ray (CXR), or microbiological tests were used in the diagnostic process. Data for CXR and microbiological tests was not available for 2006 and 2007.

Acute bronchitis was the most common diagnosis (51.6%), followed by pneumonia (24.7%), and cough (23.7%). Of the consultations, 91.6% were made during the winter season (October–March). The proportion of CRP testing in total for pneumonia, acute bronchitis, and cough increased from 55.3% in 2006 to 61.6% in 2014 (**odds ratio [OR] 1.30; 95% confidence intervals [CI] = 1.20 to 1.40; **P**<0.001). CRP was used more often when a patient was diagnosed with pneumonia (71.4%) compared to when a patient was diagnosed with acute bronchitis (61.9%; *P*<0.001). The CRP testing rate increased in the diagnostics of pneumonia from 61.3% to 77.5%, as can be seen in Figure 2 (OR 2.17; 95% CI = 1.83 to 2.59; *P*<0.001), and also increased in the diagnostics of acute bronchitis from 53.4% to 65.7% (OR 1.67; 95% CI = 1.50 to 1.86; *P*<0.001). For patients with pneumonia, the median CRP value was 62 mg/L (interquartiles, 27 and 107 mg/L), and did not change over time (*P* = 0.22); the median CRP value for patients with acute bronchitis was 11 mg/L (interquartiles, 8 and 29 mg/L).

Use of CXR, in total, changed from 6.8% in 2008 to 9.4% in 2014 (**OR 1.45; 95% CI = 1.26 to 1.66; **P**<0.001), but did not change for the diagnosis of pneumonia (*P* = 0.36), whereas it increased from 3.1% in 2008 to 5.1% in 2014 (OR 1.68; 95% CI = 1.29 to 2.18; *P*<0.001) for the diagnosis of acute bronchitis. Use of CXR also increased in the diagnosis of cough (**OR 1.47; 95% CI = 1.16 to 1.19; **P**<0.05). When CXR was performed for patients with pneumonia or acute bronchitis, CRP was analysed in 81.1% of the cases.

In total, microbiological testing increased (**OR 2.26; 95% CI = 1.82 to 2.81; **P**<0.001). This was performed in 4.5% of patients with pneumonia. The most common microbiological analysis was polymerase chain reaction for *Mycoplasma pneumonia* *e* from nasopharyngeal aspirates (2.5%), followed by nasopharyngeal swabs (0.9%) for culture of bacteria. Use of microbiological tests increased from 1.8% in 2008 to 5.1% in 2014 in the diagnostics of pneumonia (OR 2.9; 95% CI = 1.8 to 4.8; *P*<0.001) and from 1.5% to 4.1% in the diagnostics of acute bronchitis (OR 2.9; 95% CI = 2.1 to 4.1; *P*<0.001) during the same period.

**Figure 3.**
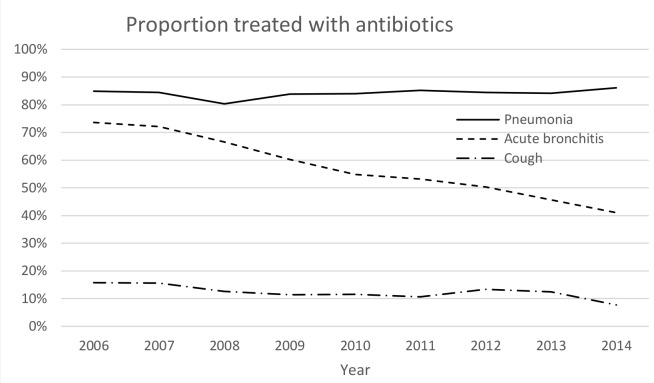
Proportions of patients aged 18–79 years with lower respiratory tract infections and cough treated with antibiotics in primary care

**Figure 4.**
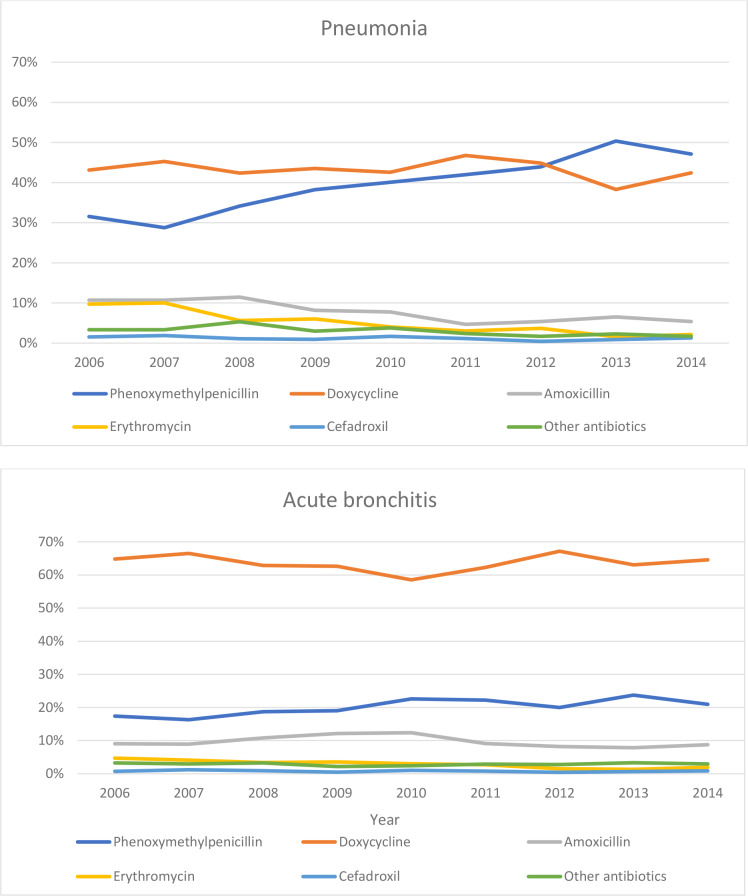
Distribution of antibiotic prescriptions for pneumonia and acute bronchitis in patients aged 18–79 years in primary care

The prevalence of different diagnoses and antibiotics prescribed during the study period are presented as *n*/1000 inhabitants per year (Table 2). The antibiotic prescription rate for patients with pneumonia was 84.3% in total and did not change (OR 1.11; 95% CI = 0.89 to 1.38; *P* = 0.38). For patients with acute bronchitis, the antibiotic prescription rate decreased from 73.6% in 2006 to 41.0% in 2014 (OR 0.25; 95% CI = 0.22 to 0.28; *P*<0.001). The significance persisted when adjusting for age, sex, and PHCC in a multiple logistic regression model. Change of antibiotic prescription rate over time is shown in Figure 3. The proportion of phenoxymethylpenicillin prescribed for patients with pneumonia increased (OR 1.9; 95% CI = 1.6 to 2.3; *P*<0.001) and amoxicillin and erythromycin decreased (*P*<0.001), whereas the proportion of doxycycline prescribed did not change (*P* = 0.74) as shown in Figure 4. When narrow-spectrum respiratory antibiotics (phenoxymethylpenicillin and amoxicillin) were prescribed for patients with pneumonia, the median CRP value was higher (72 mg/L) compared to when broad-spectrum respiratory antibiotics (doxycycline and erythromycin) were prescribed (50 mg/L) (*P*<0.001).

**Table 2.** Prevalence of pneumonia and acute bronchitis, and antibiotics prescribed in patients aged 18–79 years in primary care (*n*/1000 inhabitants each year)

	**2006**	**2007**	**2008**	**2009**	**2010**	**2011**	**2012**	**2013**	**2014**
**Pneumonia**									
Prevalence	9.7	10.3	8.6	10.4	10.9	15.8	14.0	11.3	10.5
Antibiotics prescribed	8.2	8.7	6.9	8.7	9.1	13.5	11.8	9.5	9.1
**Ac** **ute bronchitis**									
Prevalence	25.2	26.5	23.0	20.7	21.8	26.9	25.7	23.0	20.7
Antibiotics prescribed	18.6	19.1	15.3	12.5	12.0	14.3	12.9	10.5	8.5

## 



## 



## Discussion

### Summary

This register-based study on LRTIs shows that the use of CRP testing increased from 53.4% to 65.7% in the assessment of patients with acute bronchitis and from 61.3% to 77.5% for patients with pneumonia from January 2006 to December 2014. For patients with acute bronchitis, the use of CXR increased and the proportion of microbiological tests was low but increased significantly for both patients with pneumonia and patients with acute bronchitis. To the authors’ knowledge, the change in the use of diagnostic tests for the diagnosis of LRTIs in primary care over time has not been shown before.

There was a significant reduction in antibiotics prescribed for acute bronchitis, whereas the proportion of phenoxymethylpenicillin prescribed for pneumonia increased. Thus, adherence to treatment recommendations regarding assessment of LRTIs improved.

### Strengths and limitations

The large study size and the fact that data are from a whole county are strengths, minimising the risk of selection bias. The documentation of the tests performed is likely to be reliable and reflects the daily work at PHCCs. As the register is complete for primary care, there is no risk of data loss due to, for example, private surgeries. The authors decided to include the symptom diagnosis ‘cough’ to cover possible cases where physicians were uncertain of the diagnosis and patients could have been treated with antibiotics, and in that way were concealed by the diagnosis. This is a strength, and it appeared that the proportion of antibiotics prescribed for this group was low.

There are some limitations that need to be discussed. First, antibiotic prescriptions are based on those made in the EHRs. Although the authors excluded patients aged ≥80 years, there are likely to have been some younger patients who were prescribed antibiotics through a dose-dispensing system not accessible in the EHRs. Furthermore, patients with severe pneumonia were likely to have been admitted to hospital and treated with antibiotics in that context, and therefore would not have had a prescription from a PHCC. There might also have been some patients who were diagnosed with pneumonia when followed up in primary care after hospitalisation, even though visits within 6 weeks from the first consultation were excluded; therefore, they would not have had a prescription related to the contact. This could explain the somewhat low antibiotic prescription rate in the pneumonia group.

### Comparison with existing literature

Use of CRP is known to be frequent in the assessment of respiratory infections in Scandinavia, but it is not recommended in the initial judgment of pneumonia in primary care.^6,12,15^ In the present study, CRP testing increased and the frequency was high (71.4%) compared to a 2009 Swedish study by Engström *et al*, in which the corresponding frequency was only 38% in pneumonia diagnosis, and another 2016 study by Tyrstrup *et al* in which the testing rate was 60.4%, indicating that usage of point of care CRP has progressively increased.^16,17^ Earlier studies have shown that assessment of pneumonia differs between countries. For example, CRP testing is more widely used in Denmark compared to Spain, where CXR is more often used in the judgment.^11^

The incidence of pneumonia varies between 5 and 11 cases per 1000 inhabitants a year in different studies.^18,19^ The present study showed a relatively high annual incidence of pneumonia, ranging from 8.6 to 15.8/1000 inhabitants a year. The highest incidence was in 2011 when there was a *M. pneumoniae* outbreak.

When diagnostic tests are used in Swedish primary care, the PHCC not the GP is charged for the costs, which could explain the high testing frequency. Use of CRP testing has also been questioned, and one study by van Vugt *et al* showed that low values do not exclude radiographic pneumonia, whereas a study by Lagerström *et al* suggested that CRP testing can help to exclude pneumonia.^10,20^

The median CRP found in patients with pneumonia (62 mg/L) might be considered as low compared to, for example, the National Institute for Health and Care Excellence guidelines, but according to Swedish guidelines, pneumonia should be considered if CRP is >100 mg/L at the visit, or >50 mg/L after 1 week of duration.^8^ CRP is probably just a piece of the puzzle in the total judgment of the LRTI patient; however, information on the symptom duration is lacking.

Looking at the interquartiles of the CRP level for pneumonia and acute bronchitis is interesting. The lower interquartile for CRP in patients with pneumonia was 27 mg/L and the higher interquartile for CRP in patients with acute bronchitis was 29 mg/L, suggesting that CRP above 30 mg/L constitutes some kind of limit for diagnosing pneumonia, in line with the European study by van Vugt *et al*.^10^

The present study indicates that the antibiotic prescription pattern has changed over time and the proportion of prescribed phenoxymethylpenicillin, the drug of choice, has increased for the treatment of pneumonia in recent years. It is also encouraging that the antibiotic prescription rate for acute bronchitis has diminished from 73.6% to 41.0%, since guidelines do not recommend antibiotic treatment for this condition.^6^ This differs from the prescription rate for acute bronchitis in the US, where it appears to increase, and from Denmark, where the prescription rate is much lower according to a recent study by Saust *et al*.^21,22^ The efforts made by the Swedish strategic programme against antibiotic resistance (STRAMA) to illuminate the problem of resistant bacteria and to increase awareness of antibiotic resistance might have influenced the prescription pattern. It may also have increased the awareness of antibiotic resistance and contributed to increased knowledge.^23,24^

The low rate of microbiological testing is not surprising; however, the authors observed a threefold increase during the study period. The physician’s concern for *M. pneumoniae* and heightened awareness of antimicrobial resistance in general is likely to be a contributory explanation for the increase.

The use of CXR was stable for pneumonia but increased for acute bronchitis. The overall CXR rate for pneumonia was 12.4% in contrast to Saust *et al*’s Danish study, where CXR was used for 7.2% of the patients; however, that study included patients of all ages.^22^

### Implications for research and practice

In the present study, doxycycline was, in divergence with guidelines, prescribed surprisingly frequently for patients with pneumonia and was associated with lower CRP levels. An explanation could be that physicians may have suspected a probability of atypical bacterial infections, such as *M. pneumoniae* *.* This might reflect a need for further interventions in this respect, indicating that efforts made so far have not fully managed to capture any concerns of atypical bacterial infections.^25^ In the present study, the authors do not know enough about any comorbidities that might have played a role in the choice of treatment. Furthermore, the choice of antibiotics could also indicate knowledge gaps among the prescribers.^26^

Since CRP testing is increasing and the prescription rate for acute bronchitis is decreasing at the same time, this might indicate that CRP is more often used to ensure the diagnosis of acute bronchitis and, in cases of acute bronchitis, motivate the choice to refrain from prescribing antibiotics. The increasing use of both CRP and microbiological tests in diagnosing pneumonia might reflect the absence of clear diagnostic criteria and possibly a perceived need for diagnostic tests in primary care.
